# *'Special K'* and a Loss of Cell-To-Cell Adhesion in Proximal Tubule-Derived Epithelial Cells: Modulation of the Adherens Junction Complex by Ketamine

**DOI:** 10.1371/journal.pone.0071819

**Published:** 2013-08-29

**Authors:** Claire E. Hills, Tianrong Jin, Eleftherios Siamantouras, Issac K-K Liu, Kieran P. Jefferson, Paul E. Squires

**Affiliations:** 1 School of Life Sciences, University of Warwick, Coventry, United Kingdom; 2 School of Engineering, University of Warwick, Coventry, United Kingdom; 3 University Hospital of Coventry and Warwickshire, Coventry, United Kingdom; Children’s Hospital Boston, United States of America

## Abstract

Ketamine, a mild hallucinogenic class C drug, is the fastest growing ‘party drug’ used by 16–24 year olds in the UK. As the recreational use of Ketamine increases we are beginning to see the signs of major renal and bladder complications. To date however, we know nothing of a role for Ketamine in modulating both structure and function of the human renal proximal tubule. In the current study we have used an established model cell line for human epithelial cells of the proximal tubule (HK2) to demonstrate that Ketamine evokes early changes in expression of proteins central to the adherens junction complex. Furthermore we use AFM single-cell force spectroscopy to assess if these changes functionally uncouple cells of the proximal tubule ahead of any overt loss in epithelial cell function. Our data suggests that Ketamine (24–48 hrs) produces gross changes in cell morphology and cytoskeletal architecture towards a fibrotic phenotype. These physical changes matched the concentration-dependent (0.1–1 mg/mL) cytotoxic effect of Ketamine and reflect a loss in expression of the key adherens junction proteins epithelial (E)- and neural (N)-cadherin and β-catenin. Down-regulation of protein expression does not involve the pro-fibrotic cytokine TGFβ, nor is it regulated by the usual increase in expression of Slug or Snail, the transcriptional regulators for E-cadherin. However, the loss in E-cadherin can be partially rescued pharmacologically by blocking p38 MAPK using SB203580. These data provide compelling evidence that Ketamine alters epithelial cell-to-cell adhesion and cell-coupling in the proximal kidney via a non-classical pro-fibrotic mechanism and the data provides the first indication that this illicit substance can have major implications on renal function. Understanding Ketamine-induced renal pathology may identify targets for future therapeutic intervention.

## Introduction

Ketamine is a tranquilliser that has also found use as an NMDA receptor antagonist in the treatment of human bipolar disorders [1]. However, in 2006 the UK government made Ketamine a class C drug. Possessing mild hallucinogenic properties, Ketamine is rapidly replacing heroin and methamphetamine as the recreational drug of choice [2]. Cheap to buy and easily accessible, Ketamine has several street names including “Special K”, “vitamin K” and “LA Coke”. In 2008, the British Crime Survey revealed that Ketamine was the fastest growing “party drug” among 16–24 year olds and it has since been dubbed the “new ecstasy” [3]. In the UK, Ketamine boasts an estimated 125,000 users, with more young people using Ketamine in England and Wales than heroin and crack cocaine combined. As the number of users rise, serious side effects are beginning to emerge. First documented in 2007, Ketamine has been shown to injure the bladder, causing ulcers (wounds) and fibrosis (stiffening of the bladder walls and shrinkage) [4]. Patients present with multiple symptoms including incontinence, bleeding, overactive bladder and bladder shrinkage, as well as damage to both the kidneys and the ureter [5]. Despite the growing presentation of these complications, there is an acute lack of understanding for the mechanisms that underlie the pathophysiological of Ketamine, and we urgently need to investigate how this mild hallucinogenic drug scars bladder and renal tissue to impair function [6].

In adults, wound repair is commonly associated with the accumulation of scar tissue (fibrosis or sclerosis). Its effects are variable and often impaired by disease or other pathophysiological insult (e.g. diabetes/drug abuse) [7]. Fibrosis involves excess accumulation of extracellular matrix (ECM), primarily composed of collagen. As normal tissue is replaced with scar tissue, a number of phenotypic and morphological changes occur and the fibrosis ultimately results in loss of function [8]. Regardless of etiology, patients subsequently exhibit a progressive decline in organ function, a largely irreversible process that, in the case of Ketamine abuse, can lead to removal of the bladder and potential end stage renal disease. In both the bladder and kidney, early changes in protein expression/function often occur before overt fibrosis. These changes include a loss of epithelial integrity and dysregulated formation of the intercellular junction, involving, loss of epithelial E-cadherin, altered cell morphology, re-organisation of the cytoskeleton and *de-novo* expression of fibroblastic markers [9]. Cadherins have a central role in the formation of the multi-protein adherens junction, which links cell-cell contact to the actin cytoskeleton and various other signalling molecules [10]. The extracellular domain of the cell adhesion protein E-cadherin mediates ligation with neighbouring cadherins on adjacent cells [11], whilst the cytoplasmic domain binds to β-catenin linking cadherin to the actin cytoskeleton via α-catenin. The functional interaction of cadherin with F-actin, via the catenins, not only serves to increase adhesive strength of the junction but also acts as a signalling ‘node’ for proteins that influence adhesiveness &/or initiate intracellular signalling. The loss of E-cadherin mediated cell-to-cell adhesion represents a pivotal step in the transition of renal tubule cells from an epithelial phenotype to one more commonly associated with fibrosis [12]. Down-regulation of E-cadherin precedes changes in cell morphology, reorganisation of cell architecture and the subsequent gain in expression of phenotypic markers associated with renal pathology [12]–[13].

In the renal tubule, epithelial function depends on complex cell-cell interactions mediated through the adherens junction. In the current study we present data outlining early toxicological effects of Ketamine on proteins critical to formation of the adherens junction complex in the kidney. Using pharmacological manipulation, we examine potential cellular mechanisms orchestrating these changes and utilise high resolution AFM-single-cell force spectroscopy to assess the functional consequence of cell-cell tethering in Ketamine-treated cells exhibiting a loss in cadherin expression. Our data provide compelling evidence that Ketamine reduces cell-cell adhesion in epithelial cells of the proximal tubule. Translated to the *in vivo* scenario, the subsequent loss of epithelial integrity, structure and function may in part contribute to the toxicological and potential fibrotic response to Ketamine in the kidney.

## Methods

Supplies for tissue culture were purchased from Invitrogen (Paisley, UK). Immobilon P membrane was from Millipore, Watford, UK and ECL from Amersham Biosciences, Buckinghamshire, UK. A Qproteome kit was obtained from Qiagen (Sussex, UK). Antibodies were obtained from Santa Cruz (CA, USA) and R&D systems (Abingdon, UK). Ketamine, TRITC-Phalloidin and Fibronectin, were obtained from Sigma (Poole, UK), as were all other general chemicals. Anti-TGF-β1 ELISA was obtained from R&D systems.

### Model Cell Line

HK2 cells were obtained from the ATCC Bio-resource Centre (LGC Standards. Middlesex, UK). Cells (passages 18–30) were maintained in DMEM/Hams F12 (DMEM/F12) medium, supplemented with 10% fetal calf serum (FCS), glutamine (2 mM), and EGF (5 ng/ml) and cultured at 37°C in a humidified atmosphere of 5% CO_2_. Prior to treatment, cells were transferred to DMEM/F12 low glucose (5 mM) for 48 hr as described previously [14]. Cells were serum starved overnight before applying either Ketamine (0–1 mg/mL) in the presence/absence of signalling inhibitors Wortmannin (2 µM), PD98059 (10 µM), and SB203580 (1 µM) for 24 hr. Cells were pre-incubated for 30 minutes with their corresponding inhibitors prior to Ketamine application. For assessment of Smad activity HK2 cells were incubated for 0–10 minutes with Ketamine (1 mg/mL).

### MTT Assay

The 3-(4,5-Dimethylthiazol-2-yl)-2,5-diphenyltetrazolium bromide (MTT) assay is widely used for cytotoxicity assessments of pharmacological and chemical agents. Viable cells transport MTT into their mitochondria, the compound is then reduced to formazin (purple in color), and the latter is quantified colorometrically. The amount of color formed corresponds to the number of viable cells. HK2 cells were cultured in 96-well plates (5×10^3^ cells/well) in 5 mM glucose containing media for 48 hrs prior to an overnight period of serum starvation. Cells were stimulated for 24 and 48 hrs with Ketamine (0.1–1 mg) and proliferation analyzed using the MTT colorimetric assay (Roche) according to manufacturers instructions. The values were presented as a percentage of the MTT uptake that was observed as compared to control cells.

### Lactate Dehydrogenase Assay

Cell death or cytotoxicity is classically evaluated by the quantification of plasma membrane damage. Lactate dehydrogenase (LDH) is a stable enzyme, present in all cell types, and rapidly released into the cell culture medium upon damage of the plasma membrane. Therefore, LDH is a common marker used to determine cytotoxicity. HK2 cells were cultured in 96-well plates (5×10^3^ cells/well) in 5 mM glucose containing media for 48 hrs prior to an overnight period of serum starvation. Cells were stimulated for 24 and 48 hrs with Ketamine (0.1–1 mg) and Lactate dehydrogenase levels assayed using the LDH-cytoxicity assay kit II (Abcam) according to manufacturers instructions. The values were presented as a percentage of the LDH release that was observed as compared to control cells.

### Crystal Violet Assay

This is a simple assay useful for obtaining quantitative information about the relative density of cells adhering to multi-well cluster dishes. Crystal Violet stains DNA and upon solubilization, the amount of dye taken up by the monolayer can be quantitated in a plate reader. HK2 cells were cultured in 96-well plates (5×10^3^ cells/well) in 5 mM glucose containing media for 48 hrs prior to an overnight period of serum starvation. Cells were stimulated for 24 and 48 hrs with Ketamine (0.1–1 mg) and cell density determined with crystal violet staining. Briefly, media was removed and cells were fixed for 10 mins with PFA. Following a brief wash with PBS, cells were incubated for 10 mins at room temperature in a 1% Crystal Violet solution. After this time interval, all traces of dye were removed with distilled water and the stain solubilized with 1% SDS. The values were presented as a percentage of cells staining in Ketamine treated cells as compared to control cells.

### Quantification of TGF-β1

HK2 cells were cultured in 5 mM glucose containing media for 48 hrs prior to an overnight period of serum starvation. Cells were stimulated for 24 hrs with Ketamine (0.1–1 mg) under serum-free conditions and total TGF-β1 was measured by specific enzyme-linked immunosorbent assay (ELISA) of cell culture supernatant collected from growth-arrested HK2 cells. Active TGF-β1 is measured directly and latent TGF-β1 can be measured indirectly following acid activation of samples. This assay has <1% cross-reactivity for TGF-β2 and TGF-β3. TGF-β1 concentration was normalized to mg/ml of protein. Data were obtained as picograms of TGF-β1 per milliliter per mg of protein and are expressed as a percent as compared to control.

### Immunoblotting

Cytosolic proteins were prepared and separated by gel electrophoresis and electro-blotting onto Immobilon P membranes as described previously [15]. For determination of protein localization, proteins were harvested using the Qproteome cell compartment kit. Membranes were probed with specific polyclonal antibodies against anti-E-cadherin (1∶2000), N-Cadherin (1∶1000), Snail (1∶1000), Slug (1∶1000), (1∶2000), p-Smad 2 (1∶1000), p-Smad 3(1∶1000) (all R&D systems) and beta-catenin (1∶1000) (Santa Cruz).

### Immunocytochemistry

Cells at 80% confluence were fixed with 4% paraformaldehyde (PFA). Following blocking, the nuclear stain 4′, 6-diamidino-2-phenylindole, dihydrochloride (DAPI; 1 mM) was added for 3 mins. Cells were then incubated with TRITC-conjugated phalloidin (Sigma) diluted at 1∶100 in PBS-Triton for 1 hr at 25°C. Fluorescence was visualized using an Axiovert 200 fluorescence microscope (Carl Zeiss, Welwyn Garden City, UK).

### Single Cell Force Spectroscopy

Atomic Force Microscopy (AFM) Single-Cell Force Spectroscopy (CellHesion® module, JKP Instruments Germany) was used to measure cell-cell adhesion and the separation forces required to uncouple cells cultured in Ketamine as compared to control untreated cells. A single HK2 cell was bound to a cantilever using fibronectin (20 µg/ml) and poly-l-lysine (25 µg/ml) and subsequently brought into contact with an adherent cell (in a cluster of coupled cells) using a known force (1 nN). The two cells remained in contact for a defined period of time (10 sec) whilst bonding formed. The cantilever was then retracted at a constant speed (5 µm/sec) and force (nN) versus displacement (deflection of the cantilever) measured by the position of a laser beam reflected from the cantilever, until the cells were completely separated (pulling length 90 µm). Each cell-cell recording was repeated in triplicate with a 30 sec pause interval between successive measurements. Retraction recordings from multiple cells (approx. 40) in separate experiments (n = 4) were made and the maximum unbinding force (nN) and the detachment energy (fJoules) calculated.

### Analysis

Autoradiographs were quantified by densitometry using TotalLab 2003 (NonLinear Dynamics, Durham, NC USA). Where data was quantified, the non-stimulated, low glucose control condition was normalized to 100% and data from all other experimental conditions compared to this. Statistical analysis of data was performed using a one-way ANOVA test with a Tukey’s multiple comparison post-test. AFM data was analysed via *t*-test. Data are expressed as mean ± SEM, where ‘n’ denotes the number of experiments. A probability (*P*) <0.05 was taken to signify statistical significance.

## Results

### The Effect of Ketamine on Cell Viability and Cytoxicity

**Figure 1:**
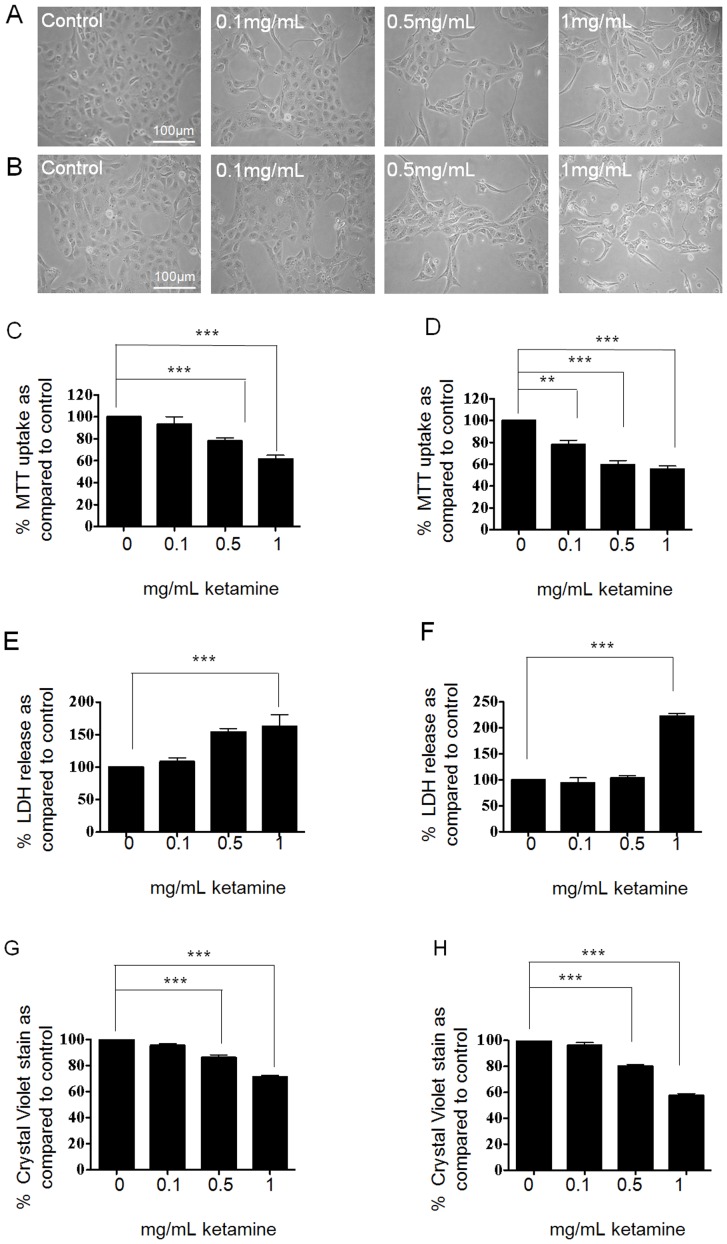
Impact of Ketamine on HK2 cell viability, as assessed by phase contrast microscopy, MTT uptake, Crystal Violet staining and LDH release. HK2 cells were cultured in 5 mM glucose containing media for 48 hrs prior to an overnight serum starvation. Cells were stimulated for 24–48 hrs with Ketamine (0.1–1 mg/mL) under serum-free conditions and morphological changes assessed (Panels A and B respectively). Ketamine evoked a concentration dependent change in cell morphology from a typical proximal tubular epithelial “cobblestone” appearance to an elongated fibroblast appearance. Cell viability was assessed by MTT uptake at 24 and 48 hrs (panels C and D respectively). Incubation with Ketamine at 0.5 and 1 mg/mL caused a significant decrease in viable cells as measured by MTT uptake. Cell membrane damage was assessed via LDH release at 24 and 48 hrs (panels E and F respectively). At both 24 and 48 hrs, Ketamine (1 mg/mL) caused a significant increase in LDH release. Finally cell density was determined by the Crystal violet assay. Ketamine evoked a reduction in cell density at 24 and 48 hrs (panels G and H respectively). The values obtained are expressed as a % of control (C). Results are representative of 3 separate experiments. Key significances are shown where **P<0.01 and ***P<0.001.

Cells were cultured in 5 mM glucose for 48 hrs prior to being serum starved overnight. Cells were either unstimulated (control) or stimulated for 24–48 hrs with Ketamine (0.1–1 mg/mL) under serum-free conditions. Phase contrast microscopy revealed that control HK2-cells exhibited a typical cobblestone morphology, characteristic of proximal tubular epithelial cells (Fig. 1, A and B). Exposure to increasing concentrations of Ketamine (0.1, 0.5 and 1 mg/mL) for 24 or 48 hrs evoked a concentration-dependent change in morphology towards an elongated fibrous phenotype. Furthermore, cells treated for 48 hrs at the highest concentration (1 mg/mL), appeared to exhibit cytosolic granulation and a reduction in cell number (Fig. 1B).

Cells stimulated for 24 and 48 hrs with Ketamine (0.1–1 mg/mL) under serum-free conditions were assessed for cell viability by MTT uptake (uptake directly correlates with the number of viable cells). At 24 hrs, incubation with Ketamine at 0.5 and 1 mg/mL significantly decreased viability by 22±4% and 39±5% respectively as compared to control (Fig. 1C, *P*<0.001, n = 3). At 48 hrs, Ketamine decreased cell viability by 22±4% (*P*<0.01), 41±3% (*P*<0.001) and 45±3% (*P*<0.001) as compared to control over the same range of concentrations (Fig. 1D, n = 3).

As a complimentary strategy to assess cytotoxicity, we used the Lactate Dehyrdogenase assay as a marker of LDH release into cell supernatant of control versus Ketamine-treated cells. To assess membrane damage, cells were stimulated for 24 and 48 hrs with Ketamine (0.1–1 mg/mL) under serum-free conditions prior to measuring LDH release. Results are expressed as a % of LDH release as compared to control cells. At 24 hrs, Ketamine (1 mg/mL) increased LDH levels by 62% to 162±17% of that under control (100%) conditions (Fig. 1E, *P*<0.001, n = 3), At 48 hrs Ketamine (1 mg/mL) significantly increased LDH levels by 140% to 240±18% as compared to control (Fig. 1F; *P*<0.001, n = 3). Release of LDH into the media suggests cell membrane damage and a concentration-dependent cytotoxic effect of Ketamine.

To further confirm the toxic role of Ketamine, we measured the number of adherent cells using a crystal violet assay. Cells were fixed and stained with crystal violet (1% w/v) at 24 and 48 hrs. Quantification of dye uptake was significantly reduced by 14±2% (*P*<0.001) and 29±1% (*P*<0.001) at 0.5 and 1 mg/mL respectively as compared to control at 24 hrs (Fig. 1G), and by 20±1% (*P*<0.001) and 43±2% (*P*<0.001) at 48 hrs over the same range of concentrations (Fig. 1H, n = 3). Based on our observations from all three strategies, subsequent analysis determined the effects of Ketamine (0.1–1 mg/mL) on cell-to-cell adhesion and adherens junction proteins over the more acute 24 hr time window.

### The Effect of Ketamine on Expression of Adherens Junction Proteins

**Figure 2:**
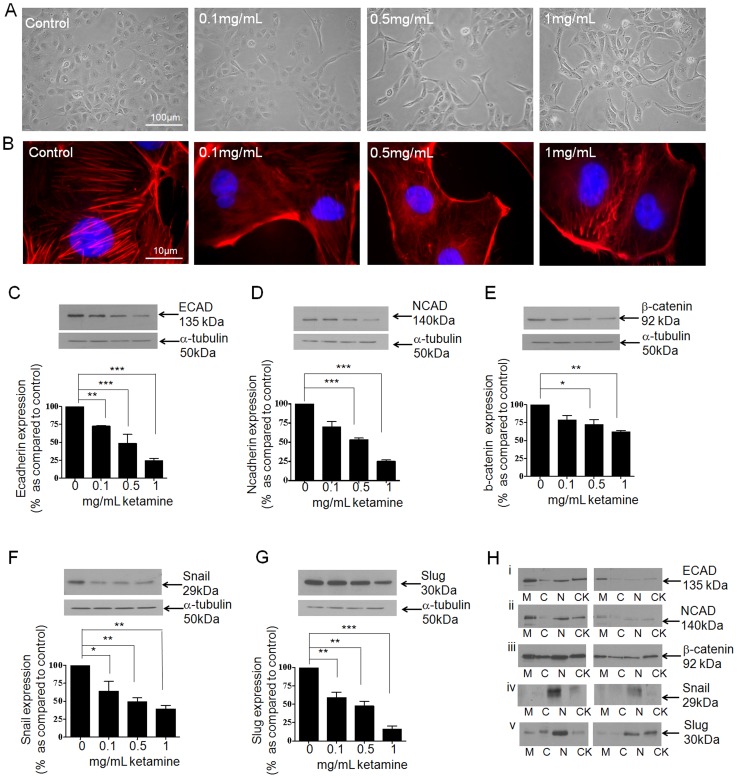
Ketamine evoked changes in AJ-protein expression in HK2-cells. To assess the effect of Ketamine on expression of key AJ proteins, HK2 cells were cultured in 5 mM glucose containing media for 48 hrs prior to overnight serum starvation. Cells were stimulated for 24 hrs with Ketamine (0.1–1 mg/mL) under serum-free conditions and morphological and phenotypic changes assessed. Phase contrast microscopy (panel A) and TRITC conjugated phalloidin (panel B) confirmed the dose dependent effects of Ketamine on cell morphology and cytoskeletal reorganization respectively. Whole cell expression of E-cadherin and its transcriptional co-repressor Snail were determined by western blotting. Ketamine decreased expression of E-cadherin (panel C) Snail (panel F) and Slug (panel G). Compartmental localisation of E-cadherin, Snail and Slug expression were determined for membrane (M), cytosol (C), nuclear (N) and cytoskeletal (CK) fractions +/− Ketamine (1 mg/mL). Ketamine altered the cellular localization of all three proteins, compared to control (panels Hi, Hiv and Hv respectively). Ketamine also down-regulated expression of N-cadherin (panel D) and β-catenin (panel E), with a loss in expression being apparent throughout the cell (panel Hii and Hiii respectively). Upper panels show representative blots for each protein and re-probed for α-tubulin as a loading control. Lower panels show mean (±SEM) densitometry data, normalised against the non-stimulated low glucose control (100%), from 3 or more separate experiments. Each lane in the representative blot corresponds to the associated bar in the graph. Key significances are shown, *P<0.05, *P<0.01, ***P<0.001.

The transition of tubular epithelial cells from a typical, cobblestone morphology to a fibrotic phenotype is commonly associated with reorganisation of cell architecture and alterations in the expression of epithelial proteins involved in adherens and tight junction formation [12]–[13]. HK2 cells were cultured in 5 mM glucose containing media for 48 hrs prior to overnight serum starvation. Cells were stimulated for 24 hrs with Ketamine (0.1–1 mg/mL) under serum-free conditions and morphological and phenotypic changes assessed. At 24 hrs, Ketamine induced a concentration-dependent change in cell morphology towards an elongated fibroblast-like phenotype (Fig. 2A). These gross morphological changes were accompanied by re-organisation of the actin cytoskeleton from a diffuse transcellular network of F-actin filaments that spanned the cytosol, into more dense peripheral stress fibres (Fig. 2B).

This architectural reorganisation intimates that Ketamine causes dysregulation of the adherens junction complex and has repercussions for the integrity of the epithelial sheet. To resolve these changes, we examined the effects of Ketamine on adherens junction proteins and their transcriptional regulators. Ketamine induced a concentration-dependent decrease in whole-cell expression of E-cadherin by 28±1% (*P*<0.01), 52±12% (*P*<0.001) and 76±3% (*P*<0.001) as compared to control at 0.1, 0.5 and 1 mg/mL respectively (n = 3, Fig. 2C). The loss in expression reflected a loss in E-cadherin from all cell compartments examined (Fig. 2Hi). Previously implicated in the differentiation of epithelial cells into fibroblast, e.g. mesenchymal cells (epithelial-mesenchymal transitions), following down regulation of expression of the adhesion protein E-cadherin [16]–[18] we next examined the effect of Ketamine on whole cell and cell-compartment expression of the transcriptional repressors Snail (Fig. 2F) and Slug (Fig. 2G). Data suggests that Ketamine down-regulated whole cell expression of Snail by 36±13% (*P*<0.05), 51±6% (*P*<0.01) and 61±4% (*P*<0.01) as compared to control (100%) and of Slug by 41±7% (*P*<0.01), 53±6% (*P*<0.01) and 84±4% (*P*<0.001) of control at 0.1, 0.5 and 1 mg/mL respectively (n = 3). Cell compartment analysis highlighted a predominant loss in expression of both these transcription factors from the nucleus (Fig. 2Hiv and Fig. 2Hv, Snail and Slug respectively). Normally exhibiting a reciprocal relationship’s with E-cadherin these data suggest a toxic, but non-lethal effect of Ketamine in cells of the proximal tubule.

The cytoplasmic domain of E-cadherin binds to β-catenin to link cell-adhesion to the actin cytoskeleton Ketamine reduced whole cell expression of β-catenin by 28±6%, 28±7% (*P*<0.05) and 39±2% (*P*<0.01) as compared to control at 0.1, 0.5 and 1 mg/mL (n = 3, Fig. 2E), an effect attributable to a loss in expression from all 4 cell compartments (Fig. 2Hiii). In the kidney, a loss in E-cadherin expression is often paralleled by a concomitant gain in the expression of neural (N)-cadherin, a mechanism commonly referred to as the cadherin switch [18]–[19]. However, in the current study, Ketamine was unable to induce this compensatory switch and Ketamine induced a concentration-dependent down-regulation in N-cadherin expression. N-cadherin expression levels decreased in response to Ketamine by to 31±7%, 47±3% (*P*<0.001) and 75±2% (*P*<0.001) as compared to control at 0.1, 0.5 and 1 mg/mL (n = 3, see Fig. 2D). Cell compartment analysis revealed that the loss in expression of N-cadherin was attributable to loss in expression at the membrane, nucleus and cytoskeleton fractions of the cell (Fig. 2Hii).

### The Effects of Ketamine are TGF-β1 Independent

**Figure 3:**
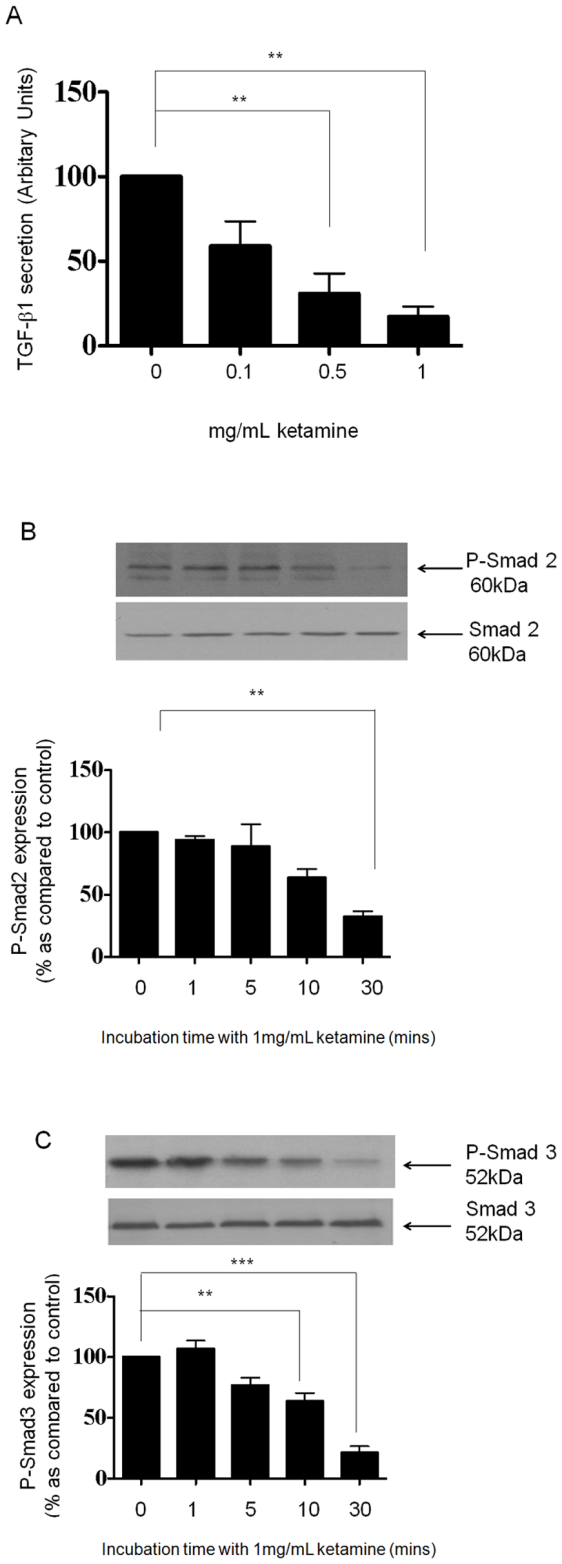
Ketamine inhibits TGF-β1 secretion and down-regulates P-Smad2 and P-Smad3 expression in HK2 cells. HK2 cells were cultured in 5 mM glucose containing media for 48 hrs prior to overnight serum starvation. Cells were stimulated for 24 hrs with Ketamine (0.1–1 mg/mL) under serum-free conditions. The supernatant was removed and TGF-β1 secretion quantified by ELISA and expressed as % TGF-β1 secretion as compared to control (panel A). Results are representative of 3 separate experiments. Key significances are shown where **P<0.01 and ***P<0.001. Whole cell expression of p-Smad2 (panel B) and p-Smad3 (panel C) were determined by immuno-blotting. HK2 cells were cultured in 5 mM glucose containing media for 48 hrs prior to overnight serum starvation. Cells were stimulated for 0–30 minutes with Ketamine (1 mg/mL) under serum-free conditions. Upper panels show representative blots for each protein and re-probed for Total Smad as a loading control. Lower panels show mean (±SEM) densitometry data, normalised against the non-stimulated low glucose control (100%), from 3 or more separate experiments. Each lane in the representative blot corresponds to the associated bar in the graph. Key significances are shown, **P<0.01, *** *P*<0.001.

It is well established that TGF-β1 is a principal mediator of fibrotic changes in the kidney. TGF-β1 modulates the expression of several epithelial cell recognition and organizational proteins, whilst contributing to the reciprocal loss of tubular epithelial cells and accumulation of interstitial fibroblasts, changes associated with declining excretory function [20]–[21]. To determine if Ketamine mediated its effects through downstream TGF-β1, we examined whether Ketamine stimulated TGF-β1 secretion (Fig. 3A) &/or regulate expression of its downstream signalling intermediates Smad2 and Smad3 (Fig. 3B and C) [22]. Cells were cultured in 5 mM glucose containing media for 48 hrs prior to an overnight serum starvation. Cells were stimulated for 24 hrs with Ketamine (0.1–1 mg/mL) under serum-free conditions and ELISA was used to measured total TGF-β1 secretion from growth-arrested HK2 cells. Ketamine significantly decreased total TGF-β1 secretion from basal control conditions (20 pg/ml) by 69±11% at 0.5 and 83±6% at 1 mg/mL as compared to control (*P*<0.01; Fig. 3A). Differences in TGF-β1 were only detected following acidification of the samples, suggesting that TGF-β1 was produced in its latent form (data not shown). Smad activity was assessed via immunoblotting. HK2 cells were cultured in 5 mM glucose containing media for 48 hrs prior to overnight serum starvation. Cells were stimulated for 0–30 minutes with Ketamine (1 mg/mL) and expression levels of p-Smad2 and p-Smad3 examined. P-Smad2 expression levels decreased by 6±3%, 12±17%, 37±7%, and 68±4% (*P*<0.01), at 1, 5, 10 and 30 minutes respectively as compared to control (Fig. 3B, n = 3). P-Smad3 expression levels were also altered in response to Ketamine with levels decreasing 24±6%, 37±7% (*P*<0.01), and 79±5% (*P*<0.001), at 5, 10, and 30 minutes respectively as compared to control (Fig. 3C, n = 3). The decrease in TGF-β1 secretion with a concomitant reduction in the associated signalling intermediaries, i.e. Smad 2 and 3, and the inability of Ketamine to induce reciprocal up-regulation of Snail and N-cadherin to coincide with a loss in E-cadherin, suggest that the cytotoxic effects of Ketamine are independent of TGF-β1.

### Ketamine Stimulates Downstream MAPK Signalling in HK2 Cells

**Figure 4:**
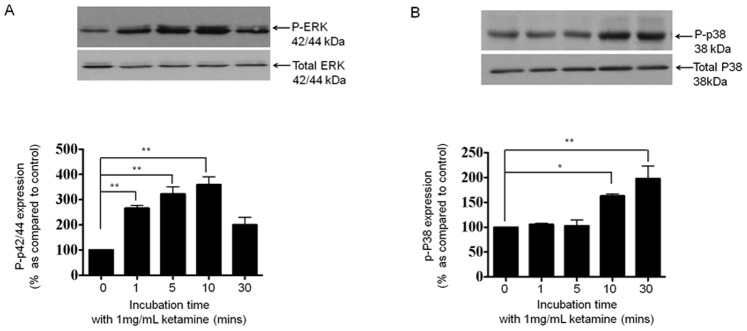
Ketamine stimulates phosphorylation of ERK and p38 MAPK. HK2 cells were cultured in 5 mM glucose containing media for 48 hrs prior to overnight serum starvation. Cells were stimulated with Ketamine (1 mg/mL) for 0–30 minutes under serum free conditions and expression of p-ERK and p-P38 assessed by immunoblotting. Upper panels show representative blots for each protein and re-probed for Total ERK and Total p38 respectively as a loading control. Lower panels show mean (±SEM) densitometry data, normalised against the non-stimulated low glucose control (100%), from 3 or more separate experiments. Each lane in the representative blot corresponds to the associated bar in the graph. Key significances are shown, **P<0.01, *** *P*<0.001.

HK2 cells were cultured in 5 mM glucose containing media for 48 hrs prior to overnight serum starvation. Cells were stimulated for 0–30 minutes with Ketamine (1 mg/mL) and expression levels of p-P38 and p-P42/44 assessed by immunoblotting (Fig. 4). P-ERK expression levels increased by 166±11% (*P*<0.01), 222±27% (*P*<0.01) and 259±30% (*P*<0.01), at 1, 5 and 10 minutes respectively as compared to control (Fig. 4A, n = 3). p-P38 expression levels were also altered in response to Ketamine with levels increasing by 63±4% (*P*<0.01), and 98±25% (*P*<0.001), at 10 and 30 minutes respectively as compared to control (Fig. 4B, n = 3).

### Signalling Cascades Regulating the Effects of Ketamine on the Adherens Junction Complex

**Figure 5:**
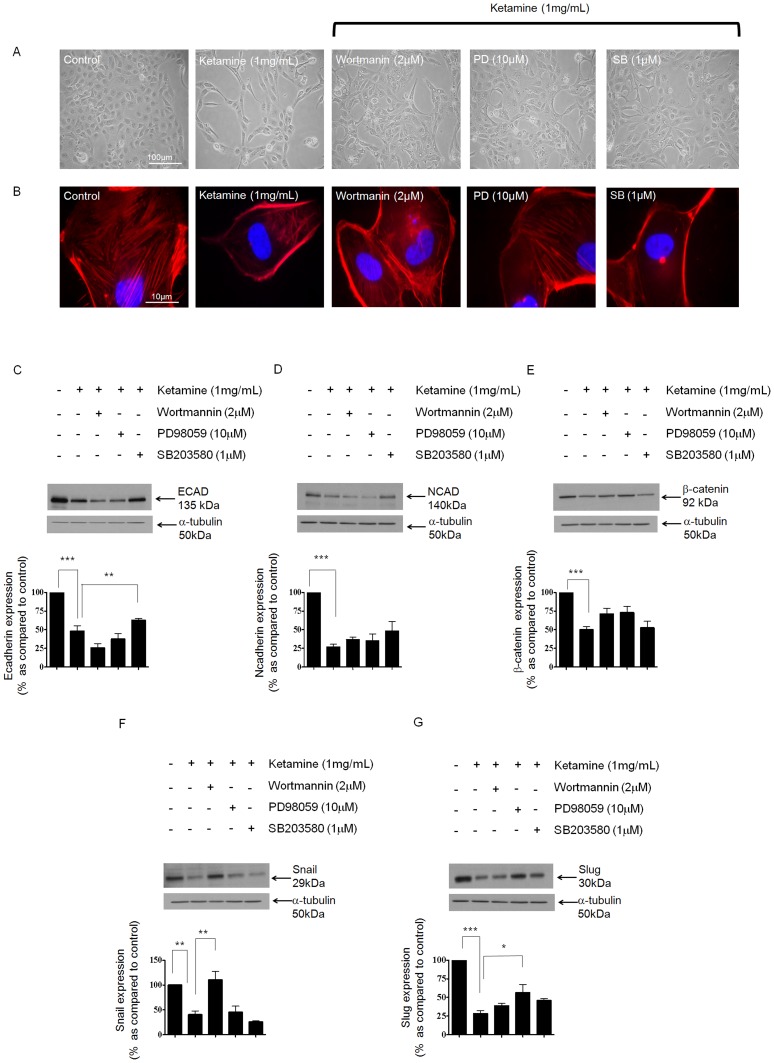
The role of PI3-K, ERK and p38 MAPK in mediating the Ketamine response. HK2 cells were cultured in 5 mM glucose containing media for 48 hrs prior to overnight serum starvation. Cells were stimulated with Ketamine (1 mg/mL) for 24 hrs in the presence or absence of Wortmannin (2 µM), PD98059 (10 µM), and SB203580 (1 µM) under serum-free conditions. Phase contrast microscopy (panel A) and TRITC conjugated phalloidin (panel B) were used to confirm if blockade of PI3-K, ERK or p38 MAPK was able to negate Ketamine-induced changes in cell morphology and cytoskeletal reorganization. HK2 cells were cultured in 5 mM glucose containing media for 48 hrs prior to overnight serum starvation. Cells were stimulated with Ketamine (1 mg/mL) for 24 hrs in the presence or absence of Wortmannin (2 µM), PD98059 (10 µM), and SB203580 (1 µM) under serum-free conditions and the expression levels of E-cadherin (panel C), N-cadherin (panel D), β-catenin (panel E), Snail (panel F) and Slug (panel G) determined by immuno-blotting. Upper panels show representative blots for each protein and re-probed for α-tubulin as a loading control. Lower panels show mean (±SEM) densitometry data, normalised against the non-stimulated low glucose control (100%), from 3 or more separate experiments. Each lane in the representative blot corresponds to the associated bar in the graph. Key significances are shown, *P<0.05, * *P*<0.01, *** *P*<0.001.

Having confirmed that Ketamine does not depend on TGF-β dependent signalling and that it can evoke activation of MAPK signalling, we examined whether the morphological and phenotypic effects of Ketamine could be arrested or reversed by pharmacological intervention with downstream inhibitors of the MAPK pathway. Cells were cultured in 5 mM glucose containing media for 48 hrs prior to an overnight serum starvation. Cells were stimulated for 24 hrs with the highest, non-lethal concentration of Ketamine (1 mg/mL) in the presence or absence of wortmannin (2 µM), PD98059 (10 µM), and SB203580 (1 µM) under serum-free conditions. Phase contrast morphology confirmed that incubation with wortmannin, PD98059, or SB203580 partly reversed gross Ketamine induced changes in cell morphology (See Fig. 5A). Qualitatively, SB203580 treated cells most closely resembled the cobblestone appearance observed under control conditions, and suggest that p38 MAPK may partially mediate the Ketamine response. Restoration of a normal cellular architecture may reflect the partial reclamation of E-cadherin expression following SB203580 treatment (see Fig. 5C). Contradictory to this, TRITC-conjugated phalloidin, suggested that the pattern of filamentous F-actin staining seen in control cells, was partially restored following treatment with the ERK inhibitor PD98059 and not SB203580 (Fig. 5B).

In order to assess changes in protein expression following inhibition of candidate signaling pathways, cells were cultured in 5 mM glucose for 48 hrs prior to an overnight serum starvation. Cells were then stimulated for 24 hrs with Ketamine in the presence or absence of wortmannin (2 µM), PD98059 (10 µM), and SB203580 (1 µM) under serum-free conditions. Whole cell expression of candidate proteins was assessed. At 24 hrs, Ketamine (1 mg/mL) decreased E-cadherin expression by 48±7% of control (Fig. 5C *P*<0.001). This effect was compounded by co-incubation with the PI3-K inhibitor wortmannin, with expression decreasing a further 36% giving a 74±5% reduction in E-cadherin expression as compared to control. Whilst wortmannin exacerbated the effect of Ketamine, PD98059 failed to significantly alter the Ketamine-evoked loss in E-cadherin. Blocking p38 MAPK improved cadherin expression in response to Ketamine (n = 3; *P*<0.01), increasing expression by 15% to 63±2% of that under control conditions and suggesting a role for p38 MAPK in regulating the effects of Ketamine on E-cadherin mediated cell adhesion (Fig. 5C).

Contrary to the data above, wortmanin completely reversed the inhibitory effects of Ketamine on Snail expression (*P*<0.01) and actually increased expression levels by 11% as compared to that observed under control control conditions (111±14%, n = 3) (Fig. 5F). As a negative regulator of E-cadherin, an increase in Snail in response inhibition of PI3-K could account for the exacerbated reduction of E-cadherin expression observed in Fig. 5C. The transcriptional repressors Snail and Slug are both situated at the core of several signaling pathways proposed to mediate EMT and are central to the regulation of E-cadherin [12]–[13]. As with Snail, Ketamine decreased Slug expression (Fig. 5G) by 72±4% as compared to control (*P*<0.001). Neither wortmannin nor SB203580 were able to significantly attenuate the inhibitory effect of Ketamine, however, inhibition of ERK regained the expression of Slug (*P*<0.05), and suggests potential involvement of ERK in regulating the effects of Ketamine over Slug expression (n = 3). In support of the data in Fig. 2, Ketamine evoked a down-regulation in both N-cadherin and β-catenin with expression levels decreasing by 73±3% and 50±4% respectively as compared to control (*P*<0.001; Fig. 5D and E). None of the inhibitors were able to negate the effects of Ketamine on these two proteins. It should be noted that treatment with inhibitors alone, did not significantly alter the expression of our five candidate proteins (see Figure S1).

### Ketamine Reduces Functional Tethering between Cells of the Proximal Tubule

**Figure 6:**
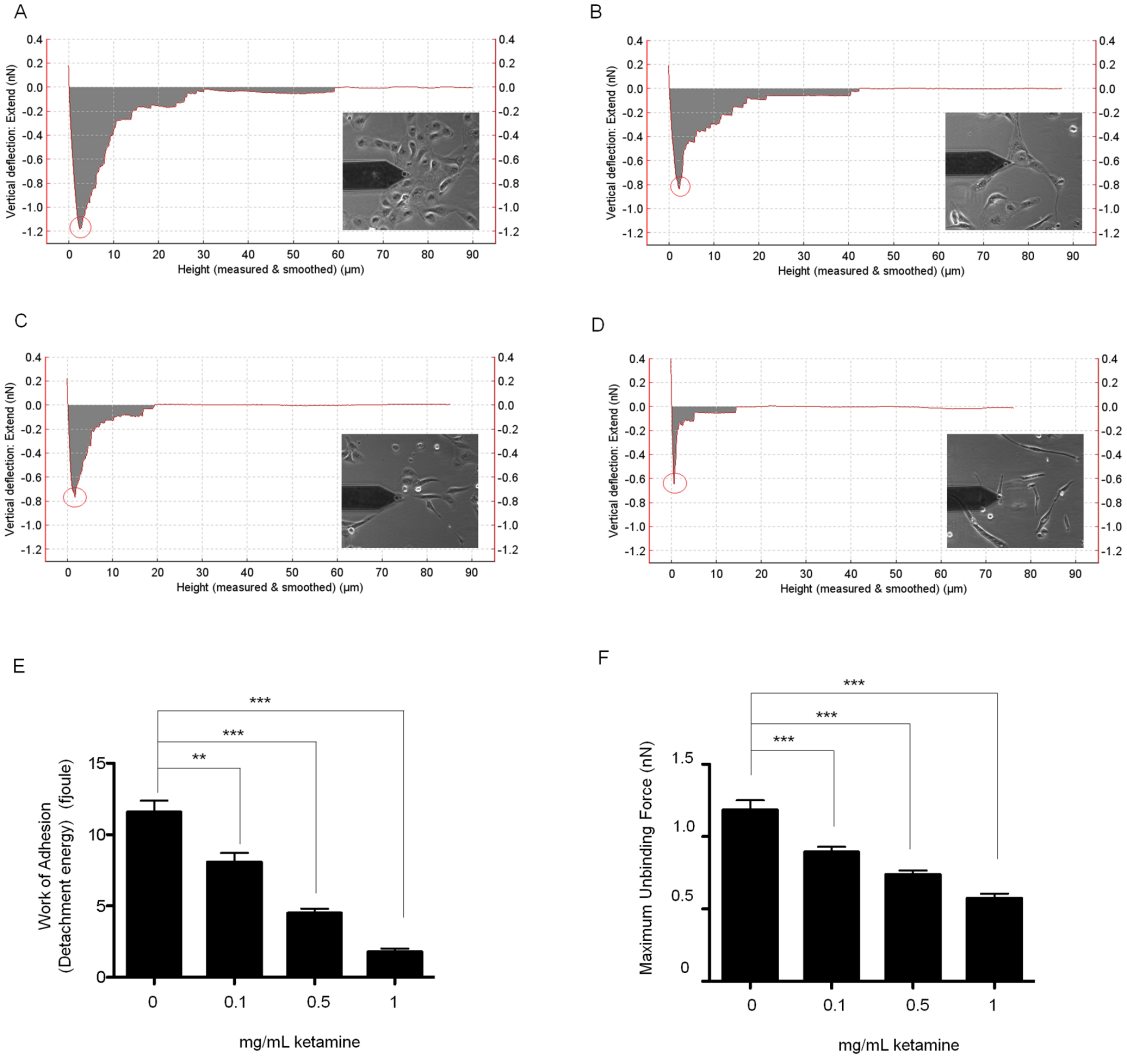
Ketamine reduces cell adhesion. AFM-single-cell force spectroscopy was used to measure the detachment energy (fJoules) and maximum unbinding force (nN) required to uncouple two HK2 cells. The energy required to separate the cells; grey area in panel A (control cells), panel B (0.1 mg/mL Ketamine treated cells), panel C (0.5 mg/mL Ketamine treated cells), and panel D (1 mg/mL Ketamine treated cells), and maximum force of detachment (red circle) was measured. The former is known as the “detachment energy” (panel E) and the later is “maximum unbinding force” (panel F). Ketamine decreased the maximum unbinding force and the work of adhesion in a dose dependent manner compared to control. Data is expressed as mean ± SEM. of multiple cells from 4 separate experiments, where key significances are shown, **** *P*<0.0001.

Atomic Force Microscopy (AFM) Single-Cell Force Spectroscopy was used to measure cell-to-cell adhesion and the separation forces and energies required to uncouple cells [23]. Prior to attachment, cells were cultured for 48 hrs under identical conditions +/− Ketamine (0.1–1 mg/mL). A single HK2 cell was bound to a cantilever and subsequently brought into contact with an adherent cell within a cluster, using a fixed force. After 10 sec, the cantilever was retracted (5 µm/sec) and force versus displacement measured until the cells were completely separated. Retraction force-displacement curves provide important information regarding the adhesion between two cells, such as the energy required to separate them (the grey area in Fig. 6A–D) and maximum force of detachment (the red circles in Fig. 6A–D). The energy required to separate two cells is normally referred to as “detachment energy” (Fig. 6E) whilst the maximum force of detachment for complete separation the “maximum unbinding force” (Fig. 6F). The retraction measurements of control versus Ketamine (0.1–1 mg/mL)-treated cells are shown in panels E & F. In each case, data is recorded from multiple cells (>10) in 4 separate experiments at each concentration. Compared to control (0 mg/mL), Ketamine evokes a concentration-dependent decrease in the maximum unbinding force by 33±6%, 41±3%, and 58±6% at 0.1 mg/mL, 0.5 mg/mL and 1 mg/mL respectively (*P*<0.001), whilst the detachment energy decreased by 32±8%, 63±7%, and 86±11% of control at 0.1 mg/mL, 0.5 mg/mL and 1 mg/mL respectively (*P*<0.001).

## Discussion

In the UK, Ketamine is fast becoming the “party drug” of choice amongst 16–24 year olds. The Independent Scientific Committee on Drugs (ISCD) found that 68% of UK clubbers had taken the drug in 2009, whilst the British Crime Survey reported an increase in users from 85,000 in 2006/07 to 113,000 in 2008/09 [2]. Although effective as a mild hallucinogen at 60 mg to 100 mg, some users are reportedly taking 5 g to 10 g a day. Stimulated by public concern about the popularity and potential harms of Ketamine, our study coincides with a recent BBC report in which the UK Home Secretary Theresa May, asked the Advisory Council on the Misuse of Drugs (ACMD) to update their advice on the abuse of ketamine. As the number of users increases, serious side effects are beginning to emerge. These effects, including bladder shrinkage, fibrosis, incontinence and bleeding, can ultimately lead to complete destruction and subsequent removal of the bladder [4]. Whilst the effects of Ketamine on the bladder are established, recent evidence suggests that damage to other tissues is also on the increase, and patients regularly present with both bladder and renal complications [24]. The escalating problem highlights an urgent need to improve our understanding of how Ketamine mediates its effects on the entire urological system.

In epithelia cells of the proximal tubule, disease-induced fibrosis commonly presents with cell atrophy, increased matrix deposition and tubulointerstitial scarring, all of which culminate in a loss of renal function [25]–[27]. The reciprocal loss of tubular epithelial cells and accumulation of interstitial fibroblasts promotes chronic fibrosis. Characteristic changes include morphological and phenotypic alterations with cytoskeletal reorganization and the down-regulation of epithelial cell adhesion molecules, such as E-cadherin. The integrity of the adherens junction is vital to maintain basic epithelial function and the loss of E-cadherin mediated cell-adhesion represents a pivotal step in early phenotypic and morphological changes observed in tubular injury [25] [28].

Changes in adherens junction proteins are central to early morphological and phenotypic alterations that precede overt signs of tissue damage. The aim of the current study was to understand of how Ketamine altered cell adhesion and cell-coupling in epithelial cells of the proximal tubule. Assessing cell morphology confirmed that Ketamine produced a concentration-dependent change in cellular architecture towards an elongated fibrotic phenotype at 24 and 48 hrs. The reduction in both MTT uptake and crystal violet staining was consistent with reduced cell viability and concomitant to an increase of lactate dehydrogenase release, confirming cytotoxicity. We confirmed that Ketamine reduced membrane expression of E-cadherin and showed for the first time that the drug decreases functional tethering between cells of the proximal tubule, a potentially catastrophic event in cells whose primary function depends on formation of a tight epithelial sheet. Whilst our previous studies in the proximal tubule suggest that a pro-fibrotic loss of E-cadherin is paralleled by an up-regulation of transcriptional repressors Snail and Slug, the effects of Ketamine appear toxic and are not mediated via classic signalling pathways [17]–[18].

The actin cytoskeleton stabilizes both tight junctions and adherent junctions and alterations in actin dynamics disrupt E-cadherin-mediated adhesion [29]–[30]. TRITC-conjugated phalloidin revealed that Ketamine redistributed F-actin in to stress fibres at the cell periphery. This data was supported by a significant reduction in expression of E-cadherin at the cell membrane, a response that, unlike classic renal fibrosis, was not paralleled by up-regulation in expression of transcription factors Snail and Slug. Furthermore, the cadherin-switch associated with EMT [18]–[19] and favouring the up-regulation of neural (N)-cadherin to replace the loss in E-cadherin, did not occur. These data suggest that the effects of Ketamine are cytotoxic, and not mediated through classic, canonical pathways usually associated with renal fibrosis.

TGF- β1 is a pro-fibrotic cytokine known to play an important role in the pathogenesis of disease-induced renal fibrosis, e.g. diabetic nephropathy [27]. Having previously shown that TGF- β1 instigates a loss in E-cadherin expression in HK2-cells, we investigated if Ketamine directly altered TGF- β1 secretion [18]. Ketamine (1 mg/mL) reduced TGF- β1 secretion to 80% of control. In renal fibrosis, TGF- β1 binds to a trans-membrane TBRII receptor and initiates several intracellular signalling cascades, including the small mothers against decapentaplegic (SMADs) and mitogen activated protein kinases (MAPK), such as extracellular regulated kinase (ERK), p38 and Jun Kinase [22]. The majority of TGF- β1 targeted genes regulated in fibrosis rely on Smad3-dependent transcriptional regulation (Brown *et al*. 2007). In the current study, Ketamine not only decreased TGF- β1 secretion but also down-regulated p-Smad2 and p-Smad3 expression at 24 hours. The failure of Ketamine to stimulate canonical TGF- β1 signalling suggests that an alternative mechanism must mediate Ketamine-evoked effects. Co-incubation of Ketamine with inhibitors of PI3-K, p38 MAPK and ERK confirmed a partial role for all three in regulating our candidate proteins. There was a counter-intuitive relationship observed between E-cadherin and Snail in response to Ketamine treatment in the presence of the PI3-K inhibitor wortmannin. Blockade of PI3-K signalling further reduced the expression of E-cadherin, an effect most likely to reflect the dramatic up-regulation Snail under the same conditions. This suggests that Ketamine down-regulates Snail expression in a PI3-K dependent manner and that blockade of this pathway restores the reciprocal relationship between E-cadherin and its transcriptional repressor. Inhibition of p38MAPK partially restored E-cadherin expression to those of control, an effect that may, in part, be responsible for the restoration of cell morphology observed with SB203580. Co-incubation of Ketamine with wortmannin failed to negate the inhibitory effects of Ketamine on Slug, whilst the ERK inhibitor, PD98059, restored expression to approximately 50% of control, indicating a downstream involvement ERK in regulating Slug. Whilst it was evident that PI3-K and ERK have a role in regulating E-cadherin, Snail and Slug, elucidating the pathway controlling N-cadherin and β-catenin expression was not clear. Ketamine reduced the expression of both these proteins and the effect could not be restored by pharmacological inhibition.

To determine how a loss in adherens junction proteins functionally affected cell-cell adhesion, we used AFM-SCFS. The AFM-single-cell force spectroscopy used in this study has a displacement actuator of longer travelling distance (up to 100 µm) which provides an excellent capability for measuring the complete force-displacement curve of cell detachment and is essential when studying large cells such as those as found in renal tubule epithelia. Retraction force-displacement curves allow us to determine the force and energy required to uncouple cells. The former is normally referred to as “adhesion force” and the latter the “detachment energy”, which can be calculated from the integration of the separation force-displacement curves, i.e. the grey area under the curve in Fig. 6A–D. Retraction force-displacement curves confirmed that 1 mg/mL Ketamine reduced the maximum unbinding force required to begin separation of two cells by 58%, whilst reducing the detachment energy required to completely separate them by 86%. The greater decrease in the detachment energy could be partly explained due to the increase in cell rigidity following Ketamine treatment, as demonstrated by re-arrangement of the cytoskeleton into peripheral stress fibres. These data suggest that it is the loss in E-cadherin expression and dissolution of the catenin/cadherin complex, which drives the detachment of cells in response to Ketamine.

In the current study, we report on Ketamine-evoked cytotoxic damage to epithelial cells of the proximal tubule. The acute effects of Ketamine are associated with early functional changes in the adherens junction complex. This loss in expression of proteins central to cell-adhesion, promotes functional disassociation of the epithelia, and is the most likely cause of early morphological and phenotypic alterations observed following Ketamine exposure. The reported changes may represent the initial basis for overt renal complications of Ketamine abuse. Whilst our studies provide novel and exciting data on the effects of this recreational drug in the proximal tubule, it is clear that this area of research demands fuller investigation. To reduce the confounding influence of the multifactorial molecular pathology that is likely to underlie Ketamine-induced renal damage within epithelia cells of the proximal tubule, we utilized the well-characterized human HK2 cell line as a minimalistic model. Despite HK2 cells having many advantages to primary tissue, the authors concede that responses are open to modification by the complexities of the *in vivo* situation. Despite this caveat, the current data provides a compelling foundation, identifying likely targets associated with early Ketamine damage, and as such, identifies future candidates for maintaining or restoring renal function in response to and from this toxic substance particularly given the catastrophic tissue damage reported for the bladder. Future work will need to extrapolate these data in to the primary scenario.

## Supporting Information

Figure S1 **The effect of Inhibitors alone on candidate protein expression in HK2 cells.** HK2 cells were cultured in 5 mM glucose containing media for 48 hrs prior to overnight serum starvation. Cells were treated for 24 hrs with Wortmannin (2 µM), PD98059 (10 µM), and SB203580 (1 µM) under serum-free conditions and the expression levels of E-cadherin (panel A), N-cadherin (panel B), β-catenin (panel C), Snail (panel D) and Slug (panel E) determined by immuno-blotting. Upper panels show representative blots for each protein and re-probed for α-tubulin as a loading control. Lower panels show mean (±SEM) densitometry data, normalised against the non-stimulated low glucose control (100%), from 3 or more separate experiments. Each lane in the representative blot corresponds to the associated bar in the graph.
(TIFF)
