# Supplementary figures and images for: 'Special K' and a Loss of Cell-To-Cell Adhesion in Proximal Tubule-Derived Epithelial Cells: Modulation of the Adherens Junction Complex by Ketamine
Source: PLoS One. 2013 Aug 29;8(8):e71819. doi: 10.1371/journal.pone.0071819 (PMC3756955; doi:10.1371/journal.pone.0071819)

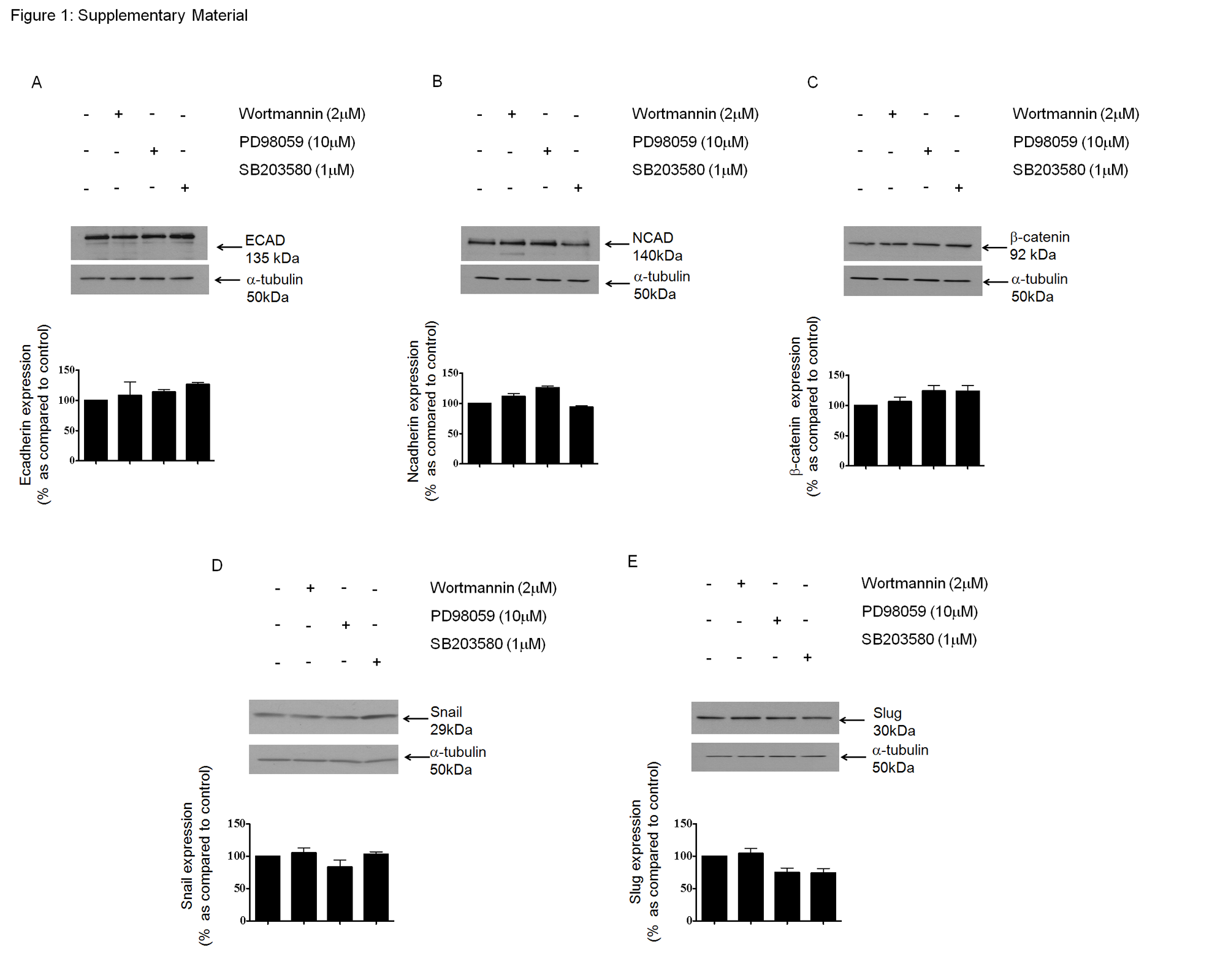

Supplement: Figure S1 — The effect of Inhibitors alone on candidate protein expression in HK2 cells. HK2 cells were cultured in 5 mM glucose containing media for 48 hrs prior to overnight serum starvation. Cells were treated for 24 hrs with Wortmannin (2 µM), PD98059 (10 µM), and SB203580 (1 µM) under serum-free conditions and the expression levels of E-cadherin (panel A), N-cadherin (panel B), β-catenin (panel C), Snail (panel D) and Slug (panel E) determined by immuno-blotting. Upper panels show representative blots for each protein and re-probed for α-tubulin as a loading control. Lower panels show mean (±SEM) densitometry data, normalised against the non-stimulated low glucose control (100%), from 3 or more separate experiments. Each lane in the representative blot corresponds to the associated bar in the graph. (TIFF) [file pone.0071819.s001.tiff]
